# Vitamin B17 Ameliorates Methotrexate-Induced Reproductive Toxicity, Oxidative Stress, and Testicular Injury in Male Rats

**DOI:** 10.1155/2020/4372719

**Published:** 2020-10-27

**Authors:** Shatha G. Felemban, Maha A. Aldubayan, Ahmad H. Alhowail, Ibtesam S. Almami

**Affiliations:** ^1^Department of Medical Laboratory Sciences, Fakeeh College for Medical Sciences, Jeddah, Saudi Arabia; ^2^Department of Pharmacology and Toxicology, College of Pharmacy, Qassim University, Saudi Arabia; ^3^Department of Biology, College of Science, Qassim University, Saudi Arabia

## Abstract

Methotrexate (MTX; 4-amino-10-methylfolic acid) is a folic acid reductase inhibitor used to treat autoimmune diseases and certain types of cancer. Testicular toxicity resulting from MTX is a significant side effect that may cause subsequent infertility. The present study was conducted to examine the ameliorating effects of vitamin B17 (VitB17) against testicular toxicity induced by MTX in male rats. A total of 50 male albino rats were equally divided into five groups [control group; vitamin B17 group (VitB17) administered VitB17 only; methotrexate group administered MTX only; cotreated group, (VitB17+MTX) and posttreated group (MTX+VitB17)]. In methotrexate group (MTX), a significant decrease was observed in body weight and the testicular weight, as well as the levels of plasma testosterone, luteinizing hormone and follicle-stimulating hormone compared with control. The sperm count, viability, morphology index, total motility, and progressive motility also decreased in MTX rats compared with control. Furthermore, the levels of reduced glutathione, catalase, and superoxide dismutase, as well as proliferating cell nuclear antigen protein expression, in the testicular tissue decreased in MTX compared with control. In addition, MTX caused a significant increase in DNA and tissue damage compared with control. However, VitB17 ameliorated these effects, indicating that it has a preventative and curative effect against MTX-induced reproductive toxicity in male rats. The protective effect of VitB17 may be associated to its antioxidant properties as it possibly acts as a free-radical scavenger and lipid peroxidation inhibitor, as well as its protective effect on the levels of GSH, SOD, and CAT.

## 1. Introduction

The testes are known to incur injury resulting from exposure to both chemotherapeutic and toxic environmental agents [1–4]. As chemotherapy drugs cannot generally distinguish between cancerous cells and noncancerous cells, toxic side effects can result [5, 6]. Although chemotherapy is effective in the treatment of different types of cancers, it causes the death of normal proliferating cells, including male germ cells [6].

Methotrexate (MTX; 4-amino-10-methylfolic acid) is a folic acid antagonist that has antineoplastic characteristics [7]. MTX achieves its chemotherapeutic effect by competing with folic acid in cancer cells, which results in a cellular folic acid deficiency and subsequent cell death. Although there are concerns regarding the toxicity of MTX [8–10], it has been used to treat certain types of cancer, such as breast, skin, neck, and lung cancers, as well as lymphoma, osteosarcoma, and acute leukemia [11]. However, this use has induced significant side effects, such as low blood cell counts, hair loss, mouth sores, and diarrhea, as well as liver, lung, nerve, and kidney damage [12, 13]. In addition, testicular damage is an important potential side effect of MTX that can lead to infertility in males [14].

Medicinal plants are good sources of exogenous antioxidants which might be considered as the new alternative approach to ameliorate pathological alterations in oxidative stress-related pathology [15–17]. Vitamin B17 (VitB17), also known as amygdalin, was first extracted from the kernels of apricots by the biochemist Ernst T. Krebs Jr. It was used to create the controversial drug Laetrile, which contains purified amygdalin. VitB17 is one of the many nitrilosides, which are natural cyanide-containing substances that are abundant in the seeds of the *Prunus* family and some members of the *Rosaceae* family, including apricots, apples, almonds, peaches, cashews, and macadamias [18, 19]. VitB17 has been used as a traditional Chinese medicine for the treatment of asthma, bronchitis, colorectal cancer, emphysema, leprosy, pain, and leukoderma [20, 21]. Due to the possibility of cyanide poisoning, Laetrile can be dangerous. In rats, the median lethal dose (LD50) of orally administered VitB17 is described as 880 mg/kg body weight, while it is 25 g/kg for intravenous injection in rats [22, 23]. A number of studies have reported that VitB17 has several pharmacological properties, including as an antioxidant, anti-inflammatory, antitussive, antiasthmatic, antiatherogenic, anticancer, and antiulcer agent, and it may inhibit or prevent fibrosis [23]. Furthermore, VitB17 from *Prunus armeniaca* seeds can induce apoptosis [24]. VitB17 can inhibit the proliferation of hepatic cancer, bladder cancer, cervical cancer, antiasthmatic, antitussive, and digestive system effects [24]. However, to the best of our knowledge, studies concerning the toxic effects of MTX on mammalian reproductive function and the counter effects of VitB17 are limited. Therefore, this study was designed to evaluate MTX-induced reproductive toxicity in male rats and the possible preventive and curative effects of VitB17.

## 2. Results

### 2.1. Toxicity

The animals in the study appeared healthy and did not show clinical signs of disease, and no mortality was recorded in the either the control group or the group receiving only VitB17 during the experiment's duration. However, various side effects were observed in animals injected with MTX, such as loss of body weight, lack of activity, weakness, and yellowish body hair. A 15 ± 3.2% mortality rate was recorded in the MTX group, while a 12 ± 1.5% mortality rate was recorded for the animal cotreated group (VitB17+MTX). Interestingly, a 18 ± 2.5% mortality rate was recorded for animals initially treated with MTX and then posttreated with VitB17 (MTX+VitB17).

The data summarized in Table 1 shows that a significant (*P* < 0.05) decrease was recorded in the relative body weight (RBW) and relative testes weight (RTW) for MTX compared with control and VitB17. However, there was a significant increase in these parameters for co- and posttreated groups compared with MTX group.

Data are expressed as *mean* ± *SE* of 5 observations. Significant difference from the control group at ^∗^*P* < 0.05. Significant difference from the methotrexate group at ^#^*P* < 0.05. *Relative* *organ* *weight* = (*Organ* *weight*/*Body* *weight*) *x* 100.

### 2.2. Effects of MTX and VitB17 on Sperm Morphometry

As would be expected due to the known toxicity of cancer drug methotrexate, the rat sperm were adversely affected in the group given methotrexate alone (group 3) compared with the control and vitamin B17 groups. All measures (sperm movement, morphology, and the number of defects among them) showed that the sperm had deteriorated after methotrexate treatment (Table 1).

However, the result in the vitamin B17 group alone (group 2) was more surprising. Rats dosed with vitamin B17 alone showed improved sperm as measured by a raised sperm count, greater movement, improved morphology, and a decrease in the number of abnormal sperm observed. This was surprising given amygdalin's known poison potential through its cyanide content (Table 1). Meanwhile, sperm abnormalities percentage exhibited significant increase in VitB17+MTX and MTX+VitB17 when compared with MTX group. On the other hand, there was a significant increase in sperm counts, sperm motility, and sperm morphological index and a significant decrease in the sperm abnormalities percentage in VitB17+MTX when compared with MTX+VitB17 groups.

### 2.3. Vitamin B17 Normalized Serum Reproductive Hormones in Methotrexate Intoxicated Rats

**Figure 1:**
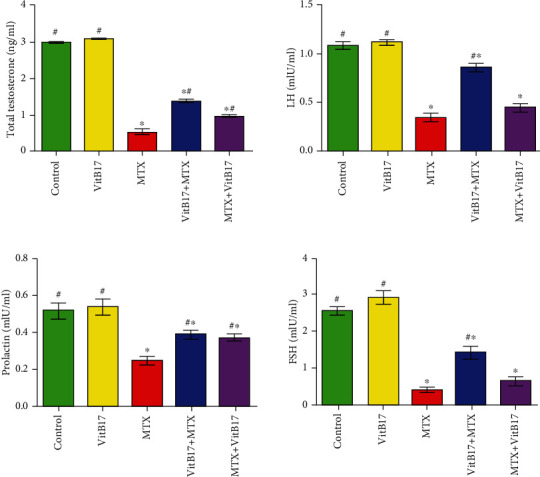
Alterations in the serum levels of total testosterone, LH, prolactin, and FSH in levels in the different groups. Vitamin B17, VitB17; methotrexate, MTX; cotreated (VitB17+MTX); posttreated, MTX+VitB17. ^∗^Significant difference (*P* < 0.05) compared with the control; ^#^significant difference (*P* < 0.05) compared with MTX.

A significant (*P* < 0.05) decrease in serum total testosterone, LH, prolactin, and FSH in treated rats with methotrexate when compared with control group (Figure 1). However, a significant increase in serum total testosterone, LH, prolactin, and FSH in VitB17+MTX and MTX+VitB17 groups when compared with methotrexate group. On the other hand, there was a significant increase in the levels of serum total testosterone, LH, prolactin, and FSH in VitB17+MTX group when compared with MTX+VitB17 group (Figure 1). Thus, vitamin B17 had potential preventive and curative effects against methotrexate-induced alteration of reproduction related hormones.

### 2.4. Effect of VitB17 on the Activities of Antioxidant Enzymes

**Figure 2:**
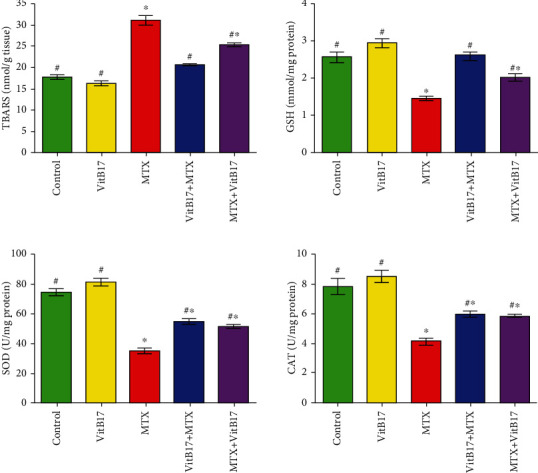
The change of testicular thiobarbituric acid-reactive substances (TBARS), reduced glutathione content (GSH) and the activities of superoxide dismutase (SOD) and catalase (CAT) activities in the different groups. Vitamin B17, VitB17; methotrexate, MTX; cotreated (VitB17+MTX); posttreated, MTX+VitB17. ^∗^Significant difference (*P* < 0.05) compared with the control; ^∗^^,#^significant difference from control and from MTX group, respectively, at *P* < 0.05.

Figure 2 shows that a significant increase was observed in testicular thiobarbituric acid reactive substances (TBARS) for G3 compared with G1 and G2, while the levels of catalase (CAT), reduced glutathione (GSH), and superoxide dismutase (SOD) significantly decreased in G3 compared with G1 and G2. On the other hand, significant decreases in testicular TBARS and significant increases in testicular CAT, GSH, and SOD were observed in both the cotreated (G4) and posttreated (G5) groups compared with G3. In addition, there was a significant decrease in testicular TBARS and significant increases in testicular CAT, GSH, and SOD in G4 compared with G5 (Figure 2).

### 2.5. Changes in Testicular DNA Fragmentation

**Figure 3:**
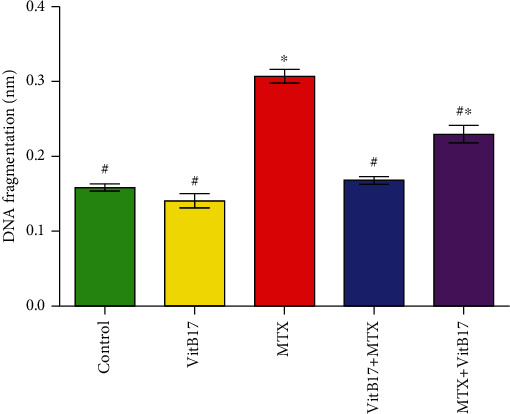
Changes of testicular DNA fragmentation in different groups. Vitamin B17, VitB17; methotrexate, MTX; cotreated (VitB17+MTX); posttreated, MTX+VitB17. ^∗^^,#^significant difference from control and from methotrexate group, respectively, at *P* < 0.05.

Figure 3 shows a significant increase in testicular DNA fragmentation in rats treated with methotrexate (MTX) compared with control rats. However, in both the cotreated (VitB17+MTX) and posttreated (MTX+VitB17) groups, there was a significant decrease in testicular DNA fragmentation compared with methotrexate (MTX). Furthermore, there was a significant decrease in testicular DNA fragmentation in cotreated (VitB17+MTX) compared with posttreated (MTX+VitB17) (Figure 3).

### 2.6. Effect of VitB17 on Testes Histopathology

**Figure 4:**
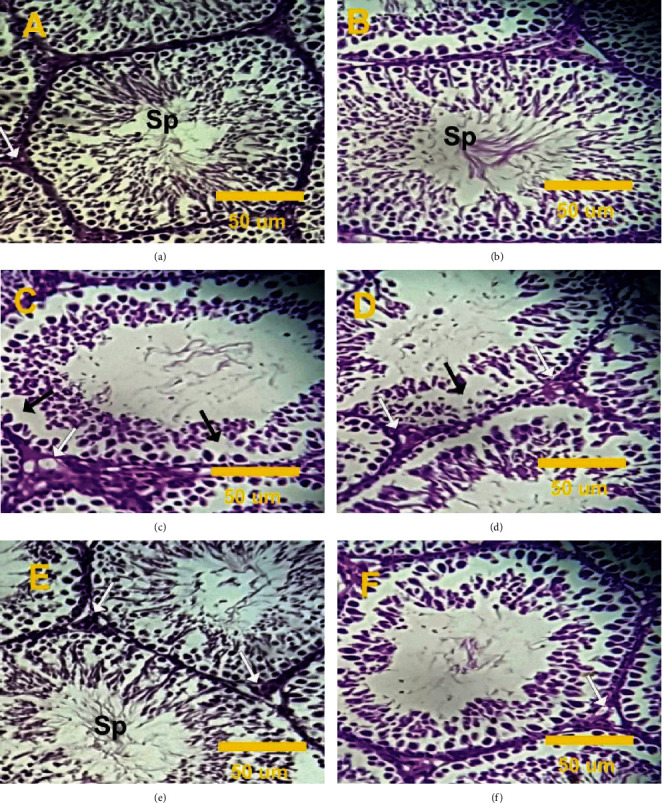
(a–f) Photomicrographs of rat testes sections in the different experimental groups stained with haematoxylin and eosin. (a, b) Testis sections in control and VitB17 groups revealed normal structure of seminiferous tubules with regular cycle of spermatogenesis and the lumen full of with sperms (Sp). (c, d) Testis sections in treated rat with methotrexate revealed disturbance and abnormal arrangement of spermatogenesis cycles (black arrows) and significance decrease in sperms and Leydig cells (white arrows). (e) Testicular section in VitB17+MTX revealed moderate increase in both sperms and Leydig cells (white arrows). (f) Testis section in MTX+VitB17 revealed mild increase in sperm numbers and few Leydig cell numbers (white arrows).

Normal histological structures were observed in the interstitial tissues (Leydig cells) and seminiferous tubules with a regular cycle of spermatogenesis in the rat testicular sections taken from control rats and rats treated with vitamin B17 only (VitB17; Figures 4(a) and (b)). In contrast, testicular sections taken from rats treated with methotrexate (MTX) revealed disturbed structures and an abnormal arrangement of the spermatogenesis cycle, with sloughing of the germ cells into the tubular lumen, marked degeneration in most of the seminiferous tubules and significance decreases of sperm and Leydig cells (Figures 4(c) and (d)). Testicular sections taken from rats in cotreated group (VitB17+MTX) revealed mild injury, with complete and moderate increases in both sperm and Leydig cells (Figure 4(e)). However, testicular sections taken from rats in post treated group (MTX+VitB17) revealed moderate injury, with mild atrophy, incomplete spermatogenesis, a decrease in Leydig cells, and a mild increase in sperm cells (Figure 4(f)).

### 2.7. Effect of VitB17 on Proliferating Cell Nuclear Antigen (PCNA) Alterations in Testes

**Figure 5:**
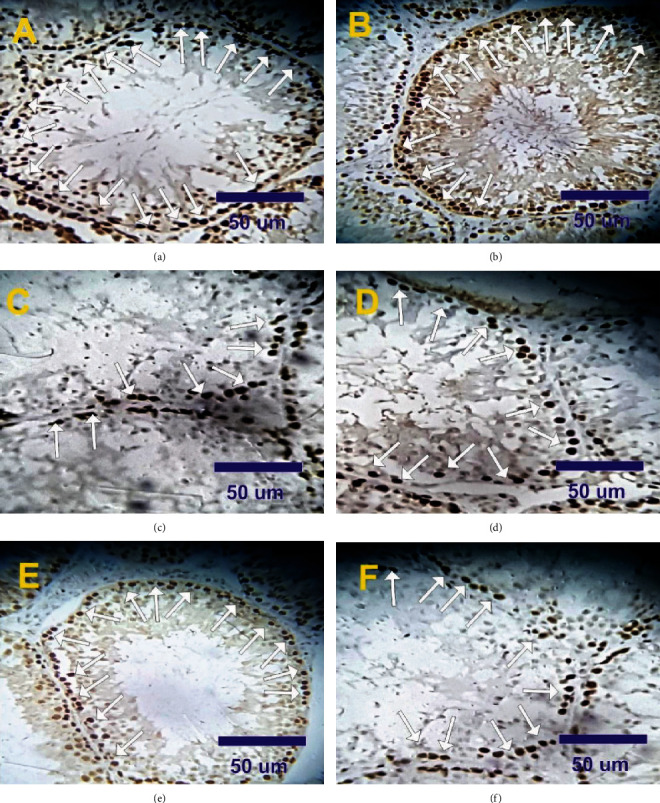
(a–f) Photomicrographs of rat testis sections stained with PCNA. (a, b) Strong positive reactions for PCNA expression (arrows) in spermatogonia in control and in treated rat with VitB17. (c, d) Mild positive reactions for PCNA expression (arrows) in treated rat with methotrexate. (e) Moderate to strong positive reactions for PCNA (arrows) in VitB17+MTX. (f) Moderate positive reactions for PCNA (arrows) in the testes of MTX+VitB17.

**Figure 6:**
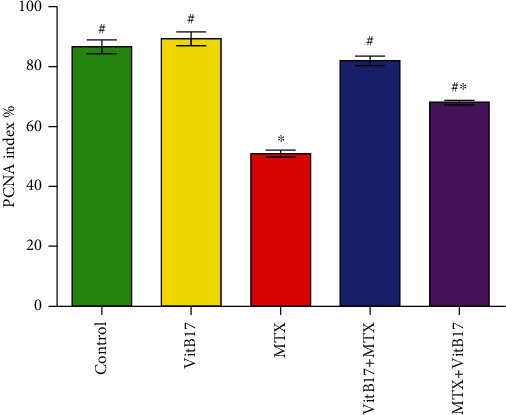
Changes of testicular PCNA-labeling index in different groups. Vitamin B17, VitB17; ,methotrexate, MTX; cotreated (VitB17+MTX); posttreated, MTX+VitB17. ^∗^^,#^significant difference from control and from methotrexate group, respectively, at *P* < 0.05.

Only the spermatogonia in control and treated rats with vitamin B17 groups showed a positive strong reaction for PCNA-ir (87.5% ± 3.5% and 91.5% ± 3.1%, respectively), while the other spermatogenic cell types showed negative reaction (Figures 5(a), 5(b), and 6). In contrast, mild positive reactions for PCNA (29.5% ± 1.7%) were observed in testicular sections in treated rat with methotrexate (Figures 5(c), 5(d), and 6). Moderate positive reactions for PCNA (74.5% ± 4.8%) were detected in the testes of (VitB17+MTX); however, mild to moderate positive reactions for PCNA (59.0% ± 3.5%) were detected in the testes of (MTX+VitB17) as compared with methotrexate group (Figures 5(e), 5(f), and 6).

## 3. Discussion

Today, there are many different kinds of chemotherapy that are used for cancer treatments. It is therefore important to search for therapies which can reduce the side effects of anticancer treatments without altering their efficacy or increasing toxicity or damage in target organs [2, 5, 7]. Vitamin B17 (VitB17) is a kind of sugar happening normally in plants, and it is a cyanogenic diglucoside found basically in fruit kernels such as apricot, peach, cashews, and macadamias [19, 20]. VitB17 has numerous pharmacological properties include antioxidant, anti-inflammatory, antitussive, and antiasthmatic activities [22]. Many research revealed that MTX induced many abnormalities and side effects during the treatments in different organs as liver and kidney toxicity [8] and in the lung and heart [5, 10]. Therefore, the current work aimed to study the possible modifying effects of vitamin B17 extract against testicular injury, sperm abnormalities, DNA damage, and proliferating PCNA alterations induced by MTX in male albino rats. Current results revealed significant decreases in the body and testicular weights of rats treated with MTX compared with the control group. The reduction in body weight may be due to disturbance in the animals' appetite and gastrointestinal tract physiology, as well as disrupted nutrient absorption occurring as a consequence of the systemic toxic effects of MTX. Additionally, the reduction in testicular weight may be due to reduction in the seminiferous tubules and the decreased number of germ cells, as well as inhibition of spermatogenesis and steroidogenic enzyme activity.

Most cases of male infertility are due to an altered sperm count or disruptions in the motility and/or morphology of sperm cells [25, 26]. Our results revealed significant decreases in the sperm count, viability, morphology index, total motility, and progressive motility in MTX rats compared with control. In contrast, significant increases in sperm abnormalities, and nonprogressive and immotile sperm were observed in MTX compared with control. The increased incidence of abnormal sperm cells and reductions in sperm density and motility are associated with increased lipid peroxidation. However, there were significant increases in the sperm count, viability, morphology index, total motility, and progressive motility after the treatment of MTX with VitB17. This situation can be explained by the fact that MTX damages cell membrane integrity by disturbing lipids and proteins within the sperm membrane. In this regard, our results agree with Padmanabhan et al., who found that weekly intraperitoneal injection of mice with MTX reduced the sperm count and increased the occurrence of sperm-head abnormalities [27]. Furthermore, Padmanabhan et al. and Yuluğ et al. [18] also found that MTX administration induced damage in the seminiferous tubules of the testes, decreased sperm count, and damaged sperm DNA [16, 18, 28]. Additionally, MTX causes defective oogenesis and spermatogenesis [14]. This effect may result from the inhibition of spermatogenesis by MTX through its impact on cell multiplication and differentiation, as it decreases the protein expression of PCNA in the spermatogonia, which is essential for DNA replication and for subsequent cell growth and proliferation [2, 27].

Our results revealed significant decreases in serum total testosterone, LH, FSH, and prolactin in MTX compared with control. The lower serum testosterone level in MTX-treated rats could be attributed to the impaired Leydig cells. This finding agrees with Sainath et al. [29] who reported that MTX-induced changes in testosterone are associated with a decreased number of LH receptors on Leydig cells [29]. Meanwhile, Badri et al. reported a decrease in steroidogenesis due to a decrease in the testosterone level as an effect of MTX after intramuscular injection [30].

Oxidative stress plays an important role in the pathogenesis of MTX-induced testicular damage [29]. It leads to damage to the structures of the testes and germ cells. Therefore, it is important to reduce cellular oxidative stress in patients receiving MTX [14]. Our results revealed a significant increase in TBARS at the same time as significant decreases in the levels of GSH, CAT, and SOD in the MTX group compared with the control group. Hence, the GSH depletion suggests that GSH may play a role in protecting cells against the adverse effects of MTX. SOD can act as a primary defense and prevents further generation of free radicals. Our results agree with Vardi et al., who reported that MTX induced testicular oxidative stress [13]. As reported in this study, CAT, SOD, and GSH levels significantly decreased in rats treated with MTX; however, VitB17 was able to modulate this effect if given concurrently or as a posttreatment to MTX. Hence, VitB17 was shown to play a protective role in alleviating the toxic effects and oxidative damage induced by MTX. Our results agree with El-Masry et al. [20] who reported that vitamin B17 was effective in controlling antioxidant enzyme activities by raising the levels of catalase GSH and SOD and decreasing the levels of MDA, H_2_O_2_, and NO, which suggests that vitamin B17 extract has free-radical scavenging and antioxidant properties. Our results revealed a significant increase in testicular DNA fragmentation in rats treated with MTX. However, as shown by the results in co- and posttreated rats, VitB17 significantly decreased testicular DNA fragmentation compared with MTX. Therefore, it can be concluded that VitB17 has a strong potential for use as a therapeutic adjuvant to MTX to prevent gonadotoxicity. In this regard, our results agree with Padmanabhan et al., who reported MTX-induced cytotoxicity and genotoxicity in the germ cells of mice [28]. Our results support this hypothesis that MTX induces biochemical, histopathological, and immunohistochemical alterations in the testes of treated rats and leads to inhibition of spermatogenesis. The effects of MTX on the testes might be due to its specific toxic effects on the target organ, rather than being a result of general toxicity. Indeed, MTX-induced testicular damage was also confirmed by the histopathological lesions observed in this study. These results suggest that MTX-induced germ cell loss may occur, in part, as a result of Sertoli cell injury-dependent alterations in the germ cell microenvironment. Our study agrees with Yuluğ et al., who reported that MTX-induced testicular damage in rats is commonly associated with spermatogenic damage, germ cell apoptosis, Leydig cell dysfunction, and testicular steroidogenic disorder [16]. Administration of VitB17 during MTX treatment also attenuated testicular damage induced by MTX, as shown by the improved sperm count and morphology, as well as the histopathological recovery, observed in co- and posttreated groups compared with MTX group. In current study, MTX-induced depletion in PCNA expression and the treatment with vitamin B17 have the ability to increase this depletion in PCNA expression. Our results agree and in the line of Mutar et al. [20] who find that vitamin B17 reduced EST induced PCNA protein expression in mice kidney tissues. Coadministration of VitB17 with MTX improved the sexual toxicity, oxidative stress, sperm count, abnormalities, and DNA damage induced by MTX. Hence, it can be stated that VitB17 alleviated the toxic effects and oxidative damage induced by MTX. The beneficial effects of vitamin B17 on semen quality may be due to increased functionality of reproductive organs, decreased levels of oxidative damage to sperm, reduced amount of energy produced by spermatozoa, decreased inflammation-induced semen impairment, and increase PCNA expression.

## 4. Materials and Methods

### 4.1. Chemicals

MTX (Methotrexate®) was obtained from Hospira UK Ltd. (United Kingdom), and VitB17 (Amygdalin) (CAS number 29883-15-6) was obtained from Cayman (Ann Arbor, MI 48108, USA) and purity ≥98%.

### 4.2. Animals

Fifty male albino rats (weighing 140 ± 10 g and aged 11–12 weeks) were bred in the animal facility at Qassim University to be used in this study. Rats were housed in Qassim University's animal house in a controlled and pathogen-free environment (25°C) with free access to water and a standard chow diet. The experiment was conducted as per the standard guidelines for animal studies after obtaining approval from the Institutional Animal Ethics Committee (approval ID number 2018-CP–16).

### 4.3. Animal Treatments

A total of 50 rats were equally divided into five groups with *n* = 10 animals per group [control group in which animals did not received any treatment; vitamin B17 (VitB17) group in which rats received VitB17 (175 mg/Kg body weight/day) (Sigma chemical Co, Germany) orally by stomach tube for four weeks according to Mutar et al. [22]; methotrexate rats group (MTX) in which rats were injected intraperitoneally with methotrexate administration (0.5 mg/kg body weight/twice a week) for four weeks according to Tousson et al. [6]; cotreated group (VitB17+MTX) in which animals injected intraperitoneally with methotrexate administration (0.5 mg/kg body weight/twice a week) and also received orally VitB17 (175 mg/Kg body weight/week) for four weeks. G5: posttreated group (MTX+VitB17) in which animals injected intraperitoneally with methotrexate administration (0.5 mg/kg body weight/twice a week) for four weeks and then treated orally with VitB17 (175 mg/Kg body weight/week) for another four weeks]. At the end of the experimental period, rats were fasted overnight and then weighed before being euthanized *via* an intravenous injection of 100 mg/kg sodium pentobarbital and subjected to a complete necropsy.

### 4.4. Sample Collection

Blood samples were individually collected from the inferior vena cava of each rat in nonheparinized glass tubes to estimate the blood parameters. Blood serum was separated by centrifugation at 4000 rpm for 10 minutes. The collected serum was stored at -20°C until analysis. Testes and epididymides were carefully removed, cleaned of adhering connective tissue in cold saline, and weighed. One testis from each pair was quickly stored at -80°C until homogenization for biochemical analysis; the other testis was fixed with neutral buffer formalin solution for histopathological and immunohistochemical examinations. Meanwhile, the epididymides were prepared for fertility evaluation (sperm count, motility, and morphology).

### 4.5. Hormone Assay

The serum level of total testosterone was measured using a solid-phase competitive chemo-luminescence enzyme immune assay (Immulite 1000; Siemens Healthcare Diagnostics, Deerfield, IL, USA) [30]. Serum levels of FSH (follicle-animating hormone), prolactin, and LH (luteinizing hormone) in sera were estimated by strong stage two-side chemo-radiance compound invulnerable measure strategies (Immulite 1000, Siemens Healthcare Diagnostics, Deerfield, IL) [31]. The assay utilizes a specific antibody or antigen-coated polystyrene beads, alkaline phosphatase conjugated reagent, and chemo-luminescence enzyme substrate Altwaijry et al. [4]. The analysis and calibration were accomplished according to manufacturer's instruction.

### 4.6. Morphometric Analysis of Sperm

The testes and epididymides were carefully removed, cleaned of adhering connective tissue in cold saline, and weighed. The epididymides were prepared for fertility evaluation that assessed the sperm count, spermatozoa motility parameters, and sperm morphology using a computer assisted semen analysis (CASA System; Germany) with an Olympus microscope (Olympus, Tokyo, Japan) [32]. A total of 200 spermatozoa from each rat were examined and individually scored normal or abnormal, according to strict sperm morphology criteria [2].

### 4.7. Tissue Preparation

Testes tissues were weighed, cut, and homogenized (10% *w*/*v*) separately in ice-cold 1.15% KCl in sodium/potassium phosphate buffer (0.01 mol/L, pH 7.4) in a Potter-Elvehjem-type homogenizer. The homogenate was centrifuged at 10,000 g for 20 minutes at 4°C, and the resultant supernatant was used for the enzyme assays.

### 4.8. Activities of Antioxidant Enzymes

To measure antioxidant enzymes, the method devised by Saggu et al. [33] was used to measure substances that reacted with thiobarbituric acid (TBARS); glutathione S-transferase (GST; EC 2.5.1.18) activity was estimated by Habig et al. [34] and Altwaijry et al. [4] utilizing para-nitrobenzyl chloride as a substrate; diminished glutathione (GSH) was estimated utilizing a strategy conceived by Moustafa et al. [35] the action of superoxide dismutase (SOD) was estimated by the technique conceived by Aldubayan et al. [36, 37].

### 4.9. DNA Fragmentation

DNA damage in testis tissue from different groups was tested by using the diphenylamine according to the method of Tousson et al. [7], which was performed to estimate the amount of DNA breakage in the tissue. The developing color of DPA was colorimetrically quantified and read with a multiwall spectrophotometer reader at wave length of 600 nm.

### 4.10. Histopathological Investigation

Testes from the different groups were fixed with 10% neutral buffered formalin solution for 24–48 hours. The fixed specimens were then dehydrated, cleaned and embedded in paraffin. Paraffin sections (5 *μ*m thick) were mounted on gelatin/chromalum-coated glass slides and stored at room temperature until further processing. Some paraffin sections were used for haematoxylin and eosin (H&E) staining *via* the routine method [38].

### 4.11. Immunohistochemical Investigation

Distribution of proliferating cell nuclear antigen immunoreactivity- (PCNA-ir) stained nuclei in kidney tissue was examined in deparaffinized sections (5 *μ*m) using Avidin–Biotin-Peroxidase immunohistochemical method (Elite-ABC, Vector Laboratories, CA, USA) with PCNA monoclonal antibody (dilution 1 : 100; DAKO Japan Co, Tokyo, Japan) [39].

#### 4.11.1. PCNA-Labeling Index

We determined the PCNA labeling index (PCNA-LI) in the PCNA immunoreactive slides by examination under a light microscope with a magnification 200x and with the help of the Image J analysis software.

### 4.12. Statistical Analyses

Results were analyzed using one-way analysis of variance (ANOVA) followed by the least significant difference (LSD) tests to compare between the different groups. Data were presented as the mean ± SEM. *P* values less than 0.05 were considered significant. All statistical analyses were performed using the SPSS Statistical Version 16 software package (SPSS® Inc., USA).

## 5. Conclusion

Administration of VitB17 had a protective and ameliorative effect against MTX-induced testicular toxicity. The protective effect of VitB17 may be associated to its antioxidant properties as it possibly acts as a free-radical scavenger and lipid peroxidation inhibitor, as well as its protective effect on the levels of GSH, SOD, and CAT.

## Figures and Tables

**Table 1:** Effects of methotrexate and/or vitamin B17 on the relative body weights (RBW), relative testes weights (RTW), sperm count, morphology index, total motility, and percent of abnormal sperms in different groups.

	Control	VitB17	MTX	VitB17+MTX	MTX+VitB17
RBW (g/100 g)	19.3^#^ ± 1.28	19.8^#^ ± 1.34	13.5^∗^ ± 1.69	16.8^∗#^ ± 1.25	15.0 ± 0.91^∗#^
RTW (g/100 g BW)	1.13^#^ ± 0.04	1.14^#^ ± 0.04	1.05^∗^ ± 0.03	1.10^#^ ± 0.06	1.09 ± 0.04^∗#^
Sperm count (million/ml)	119.5^#^ ± 4.18	131.0^#^ ± 6.75	64.5^∗^ ± 2.36	111.0^∗^ ± 7.02	95.5^#^ ± 5.60
Morphology index (%)	64.0^#^ ± 4.29	67.2^#^ ± 3.15	38.6^∗^ ± 2.96	51.45^∗^ ± 2.81	42.5^#^ ± 3.18
Total motility	76.8^#^ ± 4.83	78.2^#^ ± 5.25	16.3^∗^ ± 1.02	71.5^#^ ± 4.66	51.0^∗#^ ± 3.05
Abnormal sperms (%)	13.6^#^ ± 0.14	11.7^#^ ± 0.09	73.5^∗^ ± 4.15	21.4^#^ ± 1.45	33.7^∗#^ ± 2.04

## Data Availability

All the data are available upon request.
